# Chromosomal Instability in Hodgkin Lymphoma: An In-Depth Review and Perspectives

**DOI:** 10.3390/cancers10040091

**Published:** 2018-03-26

**Authors:** Corina Cuceu, William M. Hempel, Laure Sabatier, Jacques Bosq, Patrice Carde, Radhia M’kacher

**Affiliations:** 1 Laboratory of Radiobiology and Oncology and PROCyTOX, DRF, CEA, 91534 Paris-Saclay, France; cuceu_corina@yahoo.com (C.C.); williamhempel824@gmail.com (W.M.H.); laure.sabatier@cea.fr (L.S.); 2 Departement of Anapathology, Gustave Roussy Cancer Campus, 94805 Villejuif, France; jacques.bosq@gustaveroussy.fr; 3 Department of Hematology Gustave Roussy Cancer Campus, 94800 Villejuif, France; dr.pcarde@gmail.com; 4 Cell Environment, DNA damages R&D, Oncology section, 75020 Paris, France

**Keywords:** Hodgkin lymphoma, genomic instability, microsatellite, telomeres, micronucleus, viral infections

## Abstract

The study of Hodgkin lymphoma (HL), with its unique microenvironment and long-term follow-up, has provided exceptional insights into several areas of tumor biology. Findings in HL have not only improved our understanding of human carcinogenesis, but have also pioneered its translation into the clinics. HL is a successful paradigm of modern treatment strategies. Nonetheless, approximately 15–20% of patients with advanced stage HL still die following relapse or progressive disease and a similar proportion of patients are over-treated, leading to treatment-related late sequelae, including solid tumors and organ dysfunction. The malignant cells in HL are characterized by a highly altered genomic landscape with a wide spectrum of genomic alterations, including somatic mutations, copy number alterations, complex chromosomal rearrangements, and aneuploidy. Here, we review the chromosomal instability mechanisms in HL, starting with the cellular origin of neoplastic cells and the mechanisms supporting HL pathogenesis, focusing particularly on the role of the microenvironment, including the influence of viruses and macrophages on the induction of chromosomal instability in HL. We discuss the emerging possibilities to exploit these aberrations as prognostic biomarkers and guides for personalized patient management.

## 1. Introduction

Hodgkin lymphoma (HL) is classified into two distinct entities, classical HL (cHL), representing most cases (more than 95%), and nodular lymphocyte predominant HL (NLPHL). cHL is divided into four subtypes: nodular sclerosis (NS) (70%), mixed cellularity (MC) (20–25%), lymphocyte-rich (5%), and lymphocyte-depleted (1%) [1].

These subtypes differ in their clinical and histopathological features, especially micro-environmental features. However, all subtypes are characterized by the presence of large mononucleated or multinucleated cells with prominent nucleoli, called Hodgkin/Reed-Sternberg (HRS) cells. In the absence of recurrent chromosomal rearrangements, which normally serve both as informative diagnostic/prognostic markers and clues to deregulated gene targets located at chromosomal breakpoints [2], the cell surface marker antigens CD15 and CD30 are used to confirm the HL diagnosis after immunohistochemical staining. HRS cells represent only 1–2% of the tumor burden which includes non-malignant T cells, B cells, macrophages, granulocytes, eosinophils and stroma cells. HL represents a unique model of the interaction of a tumor cell with its microenvironment.

HL accounts for approximately 10% of all lymphomas and 1% of all cancers in industrial countries. Current treatment of HL results in a very high cure rate (80%), and thus represents the first, and one of the best, examples of successful treatment in oncology. HL also affects also young patients, and is therefore the object of intense interest amongst oncologists, cytogeneticists, and radiobiologists, insofar as lessons learned from the study of this disease and its treatment may serve as a basis for novel therapeutics and the follow-up of other cancers. Nevertheless, cohort studies of HL patients show that the survival of these patients who are essentially cured, is much lower than would be expected. The extent and nature of these morbidities and late mortalities has been the subject of numerous and detailed studies [3,4]. In addition, a substantial proportion of HL patients (20%) still experience relapsing or refractory disease, eventually progressing to death [5].

Most cases of HL were cured well before we understood the nature of the malignant cell of origin [6]. Indeed, there has been much confusion and debate on this point. Early speculation hypothesized derivation from a macrophage or histiocyte, or even a hybridized cell of uncertain origin. Kuppers et al. showed that these cells are derived from a pre-apoptotic B cell of germinal center origin with multiple somatic mutations in the clonal immunoglobulin genes [7].

Importantly, Reed-Sternberg (RS) cells represent the most conspicuous cell type in biopsy specimens and were defined as differentiated end-state HRS cells that play a pivotal role in the interaction with the tumor microenvironment in situ [8,9,10]. However, the development of these giant tumor cells has long been a subject of debate [11,12]. Recently, Rengstl et al. [13,14] demonstrated that re-fusion of daughter cells is the main route to giant HRS cell formation and also that RS cells develop neither by endomitosis nor acytokinetic mitosis. One of the major characteristics of these cells is the occurrence of complex and hyperdiploid chromosomal aberrations that reflect chromosomal instability [15,16]. Nevertheless, these abnormalities have been observed not only in HRS cells, but also in a subset of morphologically normal cells in the proximity of the HRS cells [17]. In addition, a high frequency of chromosomal aberrations and the presence of complex chromosomal rearrangements have been detected in circulating lymphocytes of HL patients before treatment [18,19]. However, the relationship between this subset of morphologically normal cells and HRS cells is not clear. The cytogenetic analysis of HL has been hampered by two major barriers: (1) the inability to grow lymph nodes in vitro and (2) the lack of a suitable animal model that can be used to understand the mechanisms underlying the chromosomal instability of HL.

Here, we discuss the implication of known mechanisms underlying chromosomal instability in HL and analyze various multistep processes in the ongoing genesis of H/RS chromosomal instability, including current hypotheses. Finally, we analyze the relationship between the mechanisms underlying genomic instability and the clinical outcome of patients.

## 2. Origin of Tumor Cells in cHL 

The scarcity of HRS cells has made it difficult to establish their origin, because they co-express the activation marker antigens of various hematopoietic lineages including B, T, dendritic, NK, and myeloid cells. Due to the lack of distinct lineage-specific markers, the activation marker antigens CD15 and CD30 are now established clinical markers for the immunohistological detection of the malignant cells in HL [20]. Until 1994, the origin and clonality of HRS cells remained obscure and many hypotheses were proposed and debated [21]. Kuppers et al. demonstrated B lineage cells as the potential origin using microdissection and single-cell PCR techniques to detect the rearrangement of immunoglobulin (Ig) genes [7,22] and concluded that germinal center (GC) B-cells are the origin of HRS cells in most cases, and that HRS cells have extinguished their normal B cell phenotype. However, clonal T cell receptor rearrangement has also been detected in a few cases, suggesting a rare subpopulation (1–2%) of HRS cells of T cell origin [23,24].

Nevertheless, several studies have suggested the existence of CD30/CD15 negative “HL cancer stem cells” or clonotypic B cells responsible for the growth and maintenance of HRS cells [25], which represent HL-initiating cells. Recently, lymphoma-specific immunoglobulin gene segments were detected in peripheral blood at initial diagnosis or during follow-up [26]. It has been well documented that mononuclear Hodgkin cells have a high proliferation potential relative to RS cells [27]. It will be informative to investigate the proliferative and clonogenic potential of CD30/CD15 negative cells isolated from HL cell lines and monitor their potential to restore the phenotype of the parental cell line. Investigation of the relationship between these clonotypic or CD30/CD15 negative B cells and HRS cells may provide not only additional information concerning the origin of the tumor cells, but may also help to define a new therapeutic strategy targeting the cancer stem cells of HL.

## 3. Advances in the Understanding of Molecular Mechanisms of the Transforming Events in cHL

Most studies in HL have focused on unraveling the molecular pathways which may play a role in the transforming events in HRS cells. Here, we focus on five important molecular pathways.

### 3.1. NF-κB Pathway

The constitutive activation of the NF-κB pathway plays an important role in HRS cells. Signal transduction through CD30, CD40, and receptor activator of NF-κB (RANK) activate the phosphorylation of IκB by the IκB kinase (IKK) complex, sustaining constitutive NF-κB signaling [28]. Various mechanisms downstream of IKK have also been shown in HL to be abnormal in HL. Mutations in the genes of the NF-κB inhibitors, IκBα and IκBε, have been found in approximately 10–20% of cases [29,30]. Genomic gains of REL, which is encoded by the TNFAIP3 gene, an inhibitor of NF-κB activity, are present in approximately 30% of HL cases [31,32], and the gene is inactivated in approximately 40% of cHL cases [33]. TNFAIP3 mutations are almost always associated with EBV infection in cHL, suggesting that TNFAIP3 inactivation and EBV infection are complementary in the pathogenesis of cHL [34].

The NF-κB pathway is activated through the gain of NF-κB-inducing kinase (NIK) in approximately 25% of the patients [21,35]. Similarly, constitutive activation of NIK in HL cell lines leads to sustained RelB signaling [36]. Other regulators of NF-κB, such as BCL3, CYLD, and TRAF3, have also been shown to be mutated, but only in rare cases of HL and in only one HL cell line [21,37,38].

### 3.2. JAK/STAT Pathway

In humans, there are four JAK and seven STAT proteins, which mediate signaling through cytokine receptors. Cytokine stimulation activates JAKs, leading to the phosphorylation of STATs. Phosphorylated STATs dimerize and translocate to the nucleus where they induce the expression of their target genes [39]. STAT3, STAT5A, STAT5B, and STAT6 have been shown to be constitutively activated in HRS cells [40,41,42,43]. Expression and activation of STAT5A and STAT5B is also increased by NF-κB activation in HRS cells [42]. In addition, IL21 is expressed in HL, leading to activation of STAT5A and STAT5B, as well as STAT3 [43]. JAK2 chromosomal gains are seen in approximately 20% of HL cases, with occasional cases of translocation [44,45]. Finally, approximately 40% of classical HL cases present inactivating mutations of SOCS1, a central important inhibitor of STAT activity [46].

Clinical studies highlight the importance of the JAK/STAT pathway in HL by demonstrating the efficacy of the blockade of the PD-1 pathway, which may be used in HL to evade immune detection. In classical HL, alterations in chromosome 9p24.1 increase the abundance of the PD-1 ligands, PD-L1 and PD-L2, and promote their induction through JAK/STAT signaling. Treatment of relapsing or refractory HL patients with the PD-1 blocking antibody, Nivolumab, resulted in an objective response for 87% of patients with 17% and 70% showing a full or partial response, respectively [47]. The association between PD-L1 protein expression and 9p24.1 alterations (polysomy, copy gain, or amplification) has been assessed in a large cohort of newly diagnosed HL patients and found to be associated with advanced stage [48] and lower EFS [49].

### 3.3. P53 Pathway

**Figure 1:**
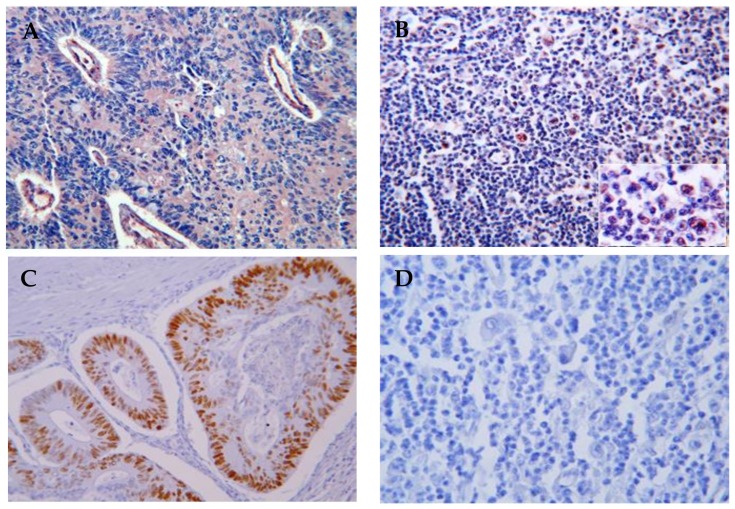
Immunohistochemistry detection of p53 proteins in HL lymph nodes (10× magnification). (**A**) p53 (mutated and wildtype) protein expression in human colon mucosa (positive control); (**B**) High p53 protein expression in HRS cells in HL lymph nodes; (**C**) Phos-p53 expression in human colon mucosa show the functionality of p53; (**D**) Lack of expression of phos-p53 in HRS cells and all HL lymph nodes, demonstrating the inhibition of p53 in HL lymph nodes.

The TP53 tumor suppressor gene can act both as a gene-specific transcription activator and transcription inhibitor. One important function of p53 is its ability to activate apoptosis. The disruption of this process can promote tumor progression and resistance to treatment [50]. It was long believed that inactivation of p53 function resulted from point mutations in exons 5 to 8, also known as hot spots (98% of all p53 mutations). Most studies have thus only superficially addressed the other regions of the gene. Alterations have been found in virtually every region of the protein, but only a handful of the most frequently occurring mutations have been studied in depth to understand their contribution to cancer progression [51]. The status of the p53 pathway in cHL remains unclear; The scarcity of identified TP53 mutations contrasts with expression levels of the p53 protein, which are often high, as demonstrated in our laboratory (Figure 1) [52]. Mutations in TP53 in cell lines derived from biopsies of cHL are rare, suggesting that they are not involved in the pathogenesis of HRS cells. However, the analysis of point mutations of the P53 tumor suppressor gene in HL is technically challenging due to the small number of HRS cells in the other tumor tissues and the use of only primer sequences of exons 5 to 8. Reevaluation of the P53 status in HL cell lines has demonstrated the presence of deletions within exon 4 in the L428 cell line and a nearly complete loss of exons 10–11 in the L1236 and exons 8–11 in the HDLM-2 cell lines. These data suggest that mutation of TP53 may be involved in the pathology of some cases of HL [53] and perhaps in the genomic instability observed during in the course of HL, as well as in the frequent occurrence of late complications, such as secondary cancer [54] or HL as a secondary event [55]. The correlation between TP53 status and high-grade progression in B-cell lymphoma has been previously established [56,57].

In summary, p53 hyper-expression and mutation may contribute to the observed chromosomal instability following profound aneuploidy and complex cytogenetic rearrangements, as well as the inter-individual heterogeneity of HL cells and clinical course of the disease.

### 3.4. ATM and ATR Pathways

In mammalian cells, ataxia-telangiectasia mutated (ATM) and ATM- and Rad3-related (ATR) kinases, members of the phosphatidylinositol 3-kinase-related kinase (PI3KK) family, play an important role in the recognition of DNA damage and the initial subsequent phosphorylation events [58,59].

The ATM gene is responsible for the ataxia-telangiectasia disorder, characterized by radiation sensitivity, immunodeficiency, and increased genomic instability [60]. The ATM gene is located on chromosome 11q22–23 and plays a major role in the activation of signaling pathways by DNA damage. Missense and truncation mutations in the ATM gene have been found in adult leukemia [61,62,63,64], as well as Mantle cell lymphoma patients [56,65,66,67,68]. Several studies have investigated the possible involvement of the ATM gene in the pathogenesis of pediatric HL [69,70]. The rare polymorphic variant of the ATM gene was observed in two series of pediatric HL, 5 of 14 (35%) [71] and 2 of 23 children (9%) [70] and was associated with a more aggressive course of the disease. Furthermore, ATM expression and function are impaired in many cases of HL [72] and several HL-derived cell lines [71,73]. The L428 cell line is characterized by aberrant downregulation of ATM activity [73] and up-regulation of FLIP protein levels [74,75]. Transient transfection of ATM was sufficient to decrease FLIP levels and induced FAS-mediated apoptosis in sensitive L428 cells [76]. This study demonstrated that targeting ATM kinase activity significantly contributes to the death receptor resistance of HL cell lines. This approach needs to be validated in subsequent studies. Of note, the screening of 52 child and adult survivors of HL who developed secondary malignancies showed no ATM mutations [77].

ATR kinases play an important role in maintaining genome integrity during DNA replication through the phosphorylation and activation of Chk1 and regulation of the DNA damage response. Only one study [78] has examined ATR gene alterations in eight HL cell lines and in seven clinical cases. Alterations of ATR were detected in six of the eight HL cell lines and in three of the seven clinical cases, most of which displayed a delay/abrogation in the repair of DNA double-strand breaks (DSBs) and single-strand breaks (SSB), as well as a defect in p53 accumulation. Further studies should investigate the putative correlation between altered ATR in genomic instability in HRS cells and the therapeutic prospect of anti-ATR antibodies.

### 3.5. FOX Pathway

FOX genes encode transcription factors which regulate basic developmental processes during embryogenesis and in the adult [79]. Several FOX genes show deregulated expression in specific malignancies, representing oncogenes or tumor suppressors [80,81]. FOXP1 and FOXM1 play contrasting roles in the pathogenesis of B-cell lymphoma, including HL. The screening of six HL cell lines for FOX gene activity by comparative microarray profiling revealed overexpression of FOXC1 and FOXD1, and reduced transcription of FOXN3, FOXO1, and FOXP1 [79]. The analysis of FOX genes in HL patient samples supported these findings. The authors proposed amplification of FOXC1 at 6p25 and a gain of the FOXR2 locus at Xp11 to be responsible for their upregulation and that activation of the TGFβ- and WNT-signaling pathways was responsible for the deregulation of FOXD1 and FOXN3. In addition, Vogel et al. provided a link between the repression of FOX1 and downregulation of PRDM1a in HL and proposed PRDM1a as a tumor suppressor in HL [82].

## 4. Mechanisms of Genomic Instability in Hodgkin Lymphoma

Two major distinct mechanisms have been described for genetic instability. The first involves distinct DNA mutations (microsatellite instability) and the second the accumulation of numerical and structural aberrations involving the gain and loss of chromosomal regions (chromosomal instability).

### 4.1. Microsatellite Instability 

Microsatellite instability (MSI) is characterized by very high mutation rates within small DNA repeat sequences (1–6 base pairs in length). This phenotype is caused by the abnormal functioning of DNA mismatch repair (MMR) genes. MSI is the most prevalent cause of hereditary non-polyposis colon cancer (HNPCC) [83,84,85], but may also result in gastric, endometrial [86], ovarian, hepato-biliary tract, urinary tract, brain, and skin cancers. In addition, MSI is a feature of immunodeficiency-related non-Hodgkin (NHL) lymphomas and HIV-related lymphomas [87,88]. The role of MSI in HL has not been extensively investigated. The studies that have been carried out were inconclusive; one study reported the presence of three of four microsatellites exhibiting interstitial rearrangements in one HL cell line [89], whereas a second found no evidence of MSI in lymph nodes of patients, suggesting that the MMR system is unlikely to contribute to the genomic instability in HL [90]. Thus, the putative involvement of MSI in HL is currently inconclusive.

### 4.2. Chromosomal Instability

The scarcity of HRS cells and technical challenges of their *in-situ* characterization, the absence of recurrent cytogenetic events, and their clonality have been a matter of debate in the past, and represent a significant challenge for the understanding, diagnosis, and treatment of HL. Indeed, only a few cHL cell lines have been approved and used worldwide [91,92]. A high level of inter-individual variation of chromosomal aberrations in HL patients accounts for the lack of a cytogenetic signature and suggests chromosomal instability.

#### 4.2.1. Chromosomal Aberrations Are Not Restricted to the HRS Cells in HL

HRS cells represent only a minority (0.1% to 1%) of the total cell population in affected lymphatic tissue. Small mononucleated malignant cells are characterized by a higher proliferation potential than bi-or polynucleated cells. Su-Ming Hsu et al. [27] demonstrated the lack of bromodeoxyuridine (BrdU), a thymidine analogue, incorporation during the replication of multinuclear cells in culture. The very low proliferative capacity of these cells was confirmed [14]. We observed no proliferation of binucleate cells in our laboratory using cytocalasin B and single-cell culture of seven HL cell lines. The lack of proliferation of binucleate cells suggests that they are likely to be end-stage cells [93,94].

Jansen et al. [17] demonstrated, that 1% to 12% of the normal-appearing small cells in the environment of HRS cells presented numerical aberrations, including frequent trisomy, using in situ hybridization in frozen or paraffin samples of HL patients. Furthermore, a higher frequency of chromosomal aberrations has been demonstrated in circulating lymphocytes of HL patients prior to any treatment [18,95,96,97]. In addition, somatic mutations in the plasma cell-free DNA of HL patients were detected [98] and proposed as a novel biomarker in cHL for both diagnosis and follow-up.

#### 4.2.2. Centrosomes, Micronuclei, and Aneuploidy

**Figure 2:**
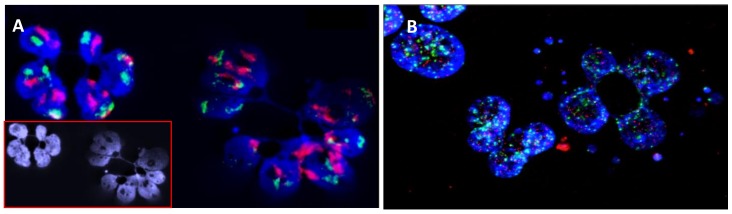
Errors of chromosome segregation and micronucleus and nucleoplasmic bridge formation in HL cells detected after four days of culture in the presence of cytocalasine B, an agent that has been most widely used to block cytokinesis and the separation of daughter cells after mitosis (40× magnification). (**A**) FISH painting of chromosome 9 (red) and 16 (green), showing the presence of defects in chromosome segregation after mitosis and the presence of nucleoplasmic bridges; (**B**) Telomere (red) and centromere (green) staining, showing the presence of multiple micronuclei with only telomere sequences (terminal deletion) and micronuclei with telomere and centromere sequences (chromosome lagging). In addition, the presence of telomere and centromere sequences in the nucleoplasmic bridge demonstrates the presence of dicentric chromosomes related to telomere fusion and the involvement of breakage-fusion-bridge cycles.

Centrosome amplification can significantly contribute to aneuploidy by favoring chromosome mis-segregation during mitosis [99] and micronucleus formation [100]. The Kelch protein Kelch-Like Domain-Containing protein 8B (KLHDC8B) [101], which is expressed during mitosis, was found to be mutated in a subset of familial and sporadic HL [102]. An association between a specific gene and centrosomal amplification, aneuploidy, and micronucleus formation in HL was established [103]. However, the precise role that centrosome amplification or duplication, micronuclei formation, and that of other nuclear abnormalities, such as nucleoplasmic bridges (NPBs) and nuclear buds (NBUDs) play in the aneuploidy and chromosomal instability observed in HL is still unclear (Figure 2).

Of note, it has been observed that prior to treatment radiation sensitivity of lymphocytes correlates with the frequency of cells containing micronuclei (MN), suggesting that high MN frequency in HL patient lymphocytes before treatment can serve as a prognostic marker for the effectiveness of radiotherapy and chemotherapy [104].

#### 4.2.3. Chromosomal Aberrations and Gene Amplification in HL 

No unique or recurrent translocation observed in malignant cells that would indicate the involvement of a putative gene or genes involved in the etiology of HL has been found, despite the large number of studies performed to identify a specific cytogenetic marker. However, these studies demonstrated the presence of a clonal population of cells with malignant characteristics. Complex chromosomal rearrangements have been frequently observed in these cells [18,105], as well as considerable aneuploidy, not only in HRS cells but also in normal-appearing lymphocytes.

On the other hand, a number of alterations common to other malignancies, including NHL, are frequently observed in HL, e.g., deletions, such as del(4q), del(6q), and del(7q), and translocations, such as t(2;5), t(14;18) [106], or t(14;19) [37], although they are not specific to HL.

Several studies analyzing copy number changes in micro-dissected HRS cells, using comparative genomic hybridization (CGH), have provided evidence of genomic imbalances, involving several regions, of either chromosome gains, including dup(2p), dup(9p), dup(17q), dup(19q), or dup(20q), or chromosome losses, including del(6q) and del(13q) [107,108]. Notably, several duplicated regions include genes known to be constitutively expressed in cHL, including gains of STAT6 (12q13), NOTCH1 (9q34), and JUNB (19p13) [108].

A novel HL susceptibility locus on chromosome 19p13.3 has been recently identified and includes the transcription factor 3 gene (known as TCF3 or E2A immunoglobulin enhancer-binding factors E12/E47), previously known to be associated with pre-B-cell acute lymphoblastic leukemia (B-ALL) [109]. In addition, NGS technologies have identified a 15–20% subset of patients with recurrent anomalies associated with high genomic instability [110].

## 5. Telomere Dysfunction in Hodgkin Lymphoma

Telomeres are dynamic nucleoprotein structures that protect the ends of chromosomes from degradation and activation of the DNA damage response. When telomeres become too short, but before genes are affected or chromosomes fuse together, cells stop dividing and undergo senescence. It is now well documented that telomere dysfunction is an important biomarker of aging and can be used in the prognosis of several diseases [111,112,113]. HRS cells show perturbed nuclear architecture, disruption of the sheltering complex, and erosion of telomeres in HL lymph nodes, as well as established HL cell lines [114,115,116], with the presence of telomere-poor ‘ghost’ nuclei [117]. Telomere sequences appear to be lost during the transition from Hodgkin to RS cells, as a significant difference has been observed between these two cell types. The organization of telomeres in Hodgkin tumor cells and in RS cells may predict treatment outcome [118].

The few studies addressing the mechanisms of telomere maintenance in HL have provided conflicting results. Decreased telomerase activity was found in 31 of 77 HL lymph nodes and higher levels in HL cell lines [119]. In contrast, Brousset et al. first demonstrated telomerase activity in HL lymph nodes [120], but subsequently showed that most cases lacked telomerase activity in a larger series (only two positive cases out of 20). A telomerase-independent mechanism for telomere maintenance in HL has been proposed, given the absence of detectable telomerase activity.

There is solid evidence that telomere length in peripheral blood lymphocytes can aid in the prediction of the development of a secondary cancer following treatment for HL [121] and it represents a risk factor for the occurrence of secondary diseases, i.e., cardiovascular disease [122,123].

These findings add to our understanding of the influence of telomeres on the occurrence of the disease, treatment outcome, and long-term effects after treatment. Telomeres represent a putative marker for individual chromosomal instability and provide the first hint for potential individualized telomere-dependent treatment [124].

## 6. Viral Infection and Chromosomal Instability in HL 

The epidemiology of HL suggests the involvement of infectious agent(s) in its pathogenesis. The role of viral infection in malignant transformation and chromosomal instability is the subject of active investigation. Numerous studies have focused on the age at first infection, from in-utero to youth and adulthood [125].

### 6.1. EBV

Epstein–Barr virus (EBV) is a human gamma-herpes virus that establishes latent infections in B lymphocytes, in which only a subset of viral genes is expressed and virus replication is suppressed [126]. The prevalence of EBV in HRS cells varies depending on the histological subtype, epidemiological factors, and age. The highest frequency is found in MC HL and the lowest in NS HL. Approximately 40% to 60% of cHL in the Western world and 90% of childhood HL are EBV positive [127]. However, the pathogenic role of EBV in HL is still not fully understood, nor the involvement of EBV in the genomic instability observed in HL. A recent study demonstrated that EBV-negative HL are characterized by more complex karyotypes than EBV-positive HL [128], despite the role of EBV nuclear antigen 1 (EBNA1) and latent membrane protein 1 (LMP1) in promoting genomic instability [129,130].

Intriguingly, short telomeres are associated with LMP1 expression in HRS cells, even in young patients [118], and EBNA1 induces loss or gain of telomere signals and promotes telomere fusion [130]. Recently, Lajoie et al. [131] demonstrated that LMP1-dependent deregulation of telomere stability and nuclear organization via shelterin downregulation, in particular TRF2, favors chromosomal rearrangements. It has been hypothesized that telomeric aggregates and ongoing breakage-fusion-bridge cycles lead to perturbed cytokinesis, resulting in multinuclearity, as proposed in EBV-associated HL [131].

Moreover, it has already been shown that patients positive for LMP1 expression have a poor prognosis, suggesting LMP1 as a new prognostic marker for HL patients [132]. Although EBV has been identified as an independent prognostic marker for poor outcome in countries endemic for EBV infection, [133], studies using pooled data obtained from different regions, including developed and underdeveloped countries, show that the presence of EBV in cHL has little effect on overall or event-free survival [134]. A further challenge is to identify the cofactors involved in the pathogenesis of HL, because most persistently infected individuals will never develop EBV-associated cancer.

### 6.2. HHV-6

One of the putative viruses involved in HL pathogenesis is Human Herpes Virus type 6 (HHV-6) [135]. Anti-HHV-6 antibodies have been detected in the serum of HL patients [135,136,137]. The implication of HHV-6 in genomic instability in HL is still obscure.

### 6.3. JCV

**Figure 3:**
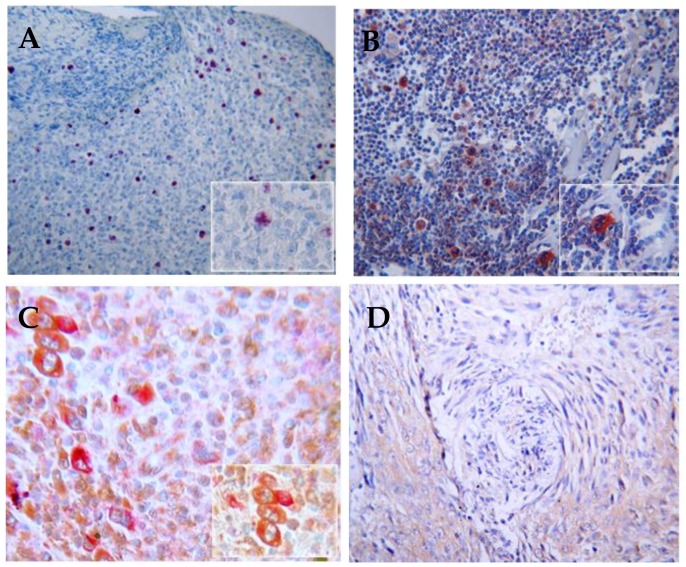
Immunohistochemical detection of JCV and EBV in HL lymph nodes (10× magnification). (**A**) Expression of LMP1 is almost always detectable in the HRS cells of EBV-associated HL (**B**) The expression of T-antigen in HL tumor cells demonstrates intense reactivity in nuclei and cytoplasm of HRS and Hodgkin tumor cells (**C**) Immunohistochemical double-labeling in HL cells demonstrates co-expression of T-antigen (Pal) and LMP1 (fast reed) in HRS cells; (**D**) Absence of immunoreactivity for anti-T-antigen and anti-LMP1 in a control sample.

Another candidate that may be involved in HL pathogenesis is the human polyomavirus, JCV, a ubiquitous virus with a seroprevalence in adults of 39–81% [138]. Most people acquire JCV in childhood or adolescence. The etiological role of JCV in lymphoma is still debated [139,140,141,142,143]. We have demonstrated that the prevalence of the JCV antibody is significantly higher in the blood of HL patients than that of NHL patients and controls (data not shown). The presence of T-antigen and agnoprotein has been detected in HL lymph nodes [144] (Figure 3). In addition, the co-activation of JCV and EBV and the presence of rogue cells [145] were observed in the peripheral blood lymphocytes of HL patients and were associated with high a risk of relapse and the mechanisms of “hit and run” [146] in HL was proposed [147].

## 7. Genomic Instability and Clinical Consequences on Prognosis and Treatment of HL Patients

Astonishingly, no correlation between chromosomal aberrations and histopathological subgroups or clinical outcomes has been investigated in HL patients. Classical cytogenetics has historically contributed little information, mostly because of the rare mitoses available from RS cells. Nevertheless, complex chromosomal rearrangements and unidentified markers in HL karyotypes have been found in advanced stages of the disease [97].

New methods to evaluate genetic lesions, such as gene expression profiling [148], comparative genomic hybridization analysis [149], micro-RNA expression profiling [150] and viral oncogene sequencing [151], and clinical outcomes of HL patients have been used and putative therapeutic targets in refractory or relapsed patients have been proposed. Further studies are needed to assess the association between genomic instability and tumor-associated macrophages in the tumor microenvironment of cHL, which have been shown to be associated with shortened PFS and OS [8,152], and PD-1/PDL-1 expression. The use of a specific cohort, such as relapsed HL patients following allogenic bone marrow transplantation [153], could help to elucidate the relationship between macrophages and malignant cells in HL [154].

The presence of telomere shortening and aggregates in HL lymph nodes have been shown to correlate with a higher risk of relapse in HL patients, especially younger patients [118]. In addition, telomere shortening in peripheral blood lymphocytes, assessed in retrospective cohorts of HL patients, has been shown to correlate with a higher occurrence of second cancers [121] and cardiovascular disease [122].

## 8. Conclusions

The mechanisms underlying genomic instability and the primary transforming events of HL are still obscure, despite the identification of the cellular origin of the malignant cells and of various cellular and molecular pathways in the pathogenesis of HL. Most of the alterations known to date may be secondary and reflect the inherent genetic instability of HRS cells. The molecular and cytogenetic characterizations of clonotypic cells [25,26] could represent the first step in determining the primary transforming event(s).

The lack of reliable animal models for HL hampers mechanistic studies of its pathogenesis, as well as the development of novel therapies. Understanding the crosstalk between malignant and non-malignant cells in the tumor microenvironment of HL could elucidate some events in this transformation such as the relationship between HL malignant cells and macrophages. The monitoring of nuclear morphology in real-time by combining time-lapse microscopy coupled with specific DNA probes could provide additional information, not only concerning the formation of RS cells (re-fusion rather than endomitosis), but also the progression to aneuploidy and the development of micronuclei. We believe that not one event, but a series of events, leads to the initiation of HL, as there is no a single cytogenetic entity in cHL.

Telomere dysfunction, loss, and deletion could be an important mechanism underlying genomic instability and oncogenesis (initiation and progression) of HL. Studying telomere dysfunction in HL families may improve our knowledge of the involvement of telomeres in the genetic susceptibility to HL. A second possible mechanism is aneuploidy and the involvement of centrosomes in the chromosome mis-segregation and micronucleus formation that lead to subsequent chromothripsis. The characterization of the link between these pathways and HL subtypes should lead to a better understanding of the mechanisms underlying this disease.
